# Matching locations is not just matching sensory representations

**DOI:** 10.1007/s00221-016-4815-1

**Published:** 2016-11-02

**Authors:** Irene A. Kuling, Marieke C. W. van der Graaff, Eli Brenner, Jeroen B. J. Smeets

**Affiliations:** Department of Human Movement Sciences, MOVE Research Institute Amsterdam, Vrije Universiteit, Amsterdam, The Netherlands

**Keywords:** Haptics, Proprioception, Sensory matching, Proprioceptive position sense, Visual localization

## Abstract

People make systematic errors when matching locations of an unseen index finger with the index finger of the other hand, or with a visual target. In this study, we present two experiments that test the consistency of such matching errors across different combinations of matching methods. In the first experiment, subjects had to move their unseen index fingers to visually presented targets. We examined the consistency between matching errors for the two hands and for different postures (hand above a board or below it). We found very little consistency: The matching error depends on the posture and differs between the hands. In the second experiment, we designed sets of tasks that involved the same matching configurations. For example, we compared matching errors when moving with the unseen index finger to a visual target, with errors when moving a visual target to the unseen index finger. We found that matching errors are not invertible. Furthermore, moving both index fingers to the same visual target results in a different mismatch between the hands than directly matching the two index fingers. We conclude that the errors that we make when matching locations cannot only arise from systematic mismatches between sensory representations of the positions of the fingers and of visually perceived space. We discuss how these results can be interpreted in terms of sensory transformations that depend on the movement that needs to be made.

## Introduction

Theories about the control of goal-directed movements try to explain things such as how one moves one’s hand to a visual target. A simple model for such a movement is the servo control: One keeps activating muscles of the arm until the hand is judged to be at the same location as the target. If the hand is not visible, the target must be judged visually and the hand haptically (in this paper, we make no distinction between haptic judgments based on cutaneous, proprioceptive and efferent signals). If movements are controlled in this manner, and given sufficient time to move, systematic errors in reaching the target (which we will refer to as matching errors) logically imply that there is some kind of mismatch between the senses. Such matching errors are frequently reported, for instance when moving the finger of one hand to the position of the finger of the other hand (Von Hofsten and Rösblad 1988; Van Beers et al. 1996; Haggard et al. 2000). These errors have a sensory (and not motor) basis as they are present for slow movements and also present if participants explicitly have to indicate when no more mismatch is sensed (Kuling et al. 2013). Although within subjects these matching errors are systematic and consistent over time (Kuling et al. 2016), there are large differences between subjects, i.e., the errors are idiosyncratic. Moreover, although the errors are generally similar across the workspace, they are larger and more variable for localization further away from the body (Van Beers et al. 1998). According to the above reasoning, these idiosyncratic systematic errors could be considered to represent systematic sensory mismatches that can differ between subjects.

The origin of such sensory mismatches is unclear. There are three (not mutually exclusive) possible origins. A first possibility is that the mismatches are due to systematic, modality-specific misjudgments of positions in space, perhaps arising from systematic differences between the ways in which the positions of the fingers and visual positions are represented. If so, we can consider them to be mismatches between sensory maps of our surrounding. We use the term “map” to refer to any representation of space, irrespective of the reference frame within which positions on the map are described. The maps could simply be shifted relative to each other, but they could also differ in scale or orientation, or have modality-specific complex deformations such as might arise from scaling binocular disparities to help obtain a visual map. All these cases would lead to similar systematic mismatches across trials, regardless of how the match is achieved (both in terms of posture and in terms of the actions involved). Deviations across trials could arise from sensory or motor variability, but the systematic component is the result of the way in which positions in space are coded for the different modalities and possibly effectors.

A second possibility is that mismatches arise from systematic errors in sensing joint angles. If so, matching errors will not only depend on the position of the tip of the finger, but on the posture of the arm as well, because systematic errors in judging joint angles will give rise to different errors when the posture of the arm is different, even if the target position for the finger is not different. Consequently, it will be impossible to describe matching errors as aligning sensory maps of the surrounding.

A third possibility is that systematic mismatches do not only arise from modality-specific misjudgments of positions in space and systematic errors in sensing joint angles, but also on the way in which the final position and posture is achieved, perhaps because it depends on the specific reference frame transformation that is required for the task (McIntyre and Lipshits 2008). For instance, it might arise when transforming information about the position of a visual target, which is probably represented in a gaze-centered reference frame, to a desired posture in a body-centered haptic reference frame. If the systematic errors arise during the transformations, rather than being present in the representations themselves, they might even depend on which of two positions is matched to the other. Which of these three explanations (map-based, posture-based or transformation-based) gives the most comprehensive description?

According to the map-based explanation, the reason for there being mismatches between the hands when matching the position of one hand with the other hand is that the haptic maps of our two hands are not completely aligned. This is not implausible, because it is well known that the dominant and non-dominant hand differ in many respects. For instance, Sainburg and colleagues have shown that the dominant and non-dominant hand are controlled differently (Sainburg and Kalakanis 2000; Sainburg and Schaefer 2004). Moreover, the origins of the maps might also differ, which would result in mismatches if there are any systematic deformations of space, even if the deformations are the same for the two arms. However, differences between the maps may not be the only or even the main source of mismatches.

There is some evidence that matching errors differ for different postures, which speaks against the map-based explanation. Many of the above-mentioned mismatches could be due to differences in posture, rather than differences between the arms, because subjects were generally asked to match the positions of the two index fingers by aligning them at opposite sides of a horizontal surface, so that the postures of the two arms and hands were quite different. Rossetti et al. (1994) explicitly examined how the posture of the arm influences such position-matching errors. Subjects had to match the position of their right index finger with their left index finger. The right index finger was placed at one of four positions on the table, including ones that involved extreme, uncomfortable, joint angles. The variability in the matches increased with the discomfort of the posture, as well as with the distance from the body. Besides the differences in variability between the postures, there were also significant systematic differences between the matching errors. Although this seems to support the posture-based explanation, it might be an artifact of the uncomfortable postures that were chosen by the experimenters. In the present study, we therefore tested whether a similar effect of posture also occurs for more comfortable postures.

According to both the map-based and posture-based explanations, the systematic matching errors are caused by systematic inconsistencies between two (or more) judgments about one’s position relative to the surrounding space. This could be due to errors in the haptic judgment of the position of the hands, but it could also be due to misjudging visually perceived positions. If one were to ask subjects to align an index finger with a visual target, instead of to align it with the other index finger, the proprioception of the arm in question would contribute to the visuo-haptic matching error in the same way as it does when aligning the two index fingers. The contribution of proprioception of the other arm to the matching error would be replaced by errors in visual localization of the target (Sousa et al. 2010). Matching the hand’s position to that of a visual target does indeed lead to systematic matching errors (Van Beers et al. 1998; Smeets et al. 2006; Rincon-Gonzalez et al. 2011; Kuling et al. 2013; Van der Kooij et al. 2013; Kuling et al. 2015), and such errors are also consistent over long periods of time (Kuling et al. 2016). Following this reasoning, knowing the systematic errors that someone makes when matching one index finger to the other, and knowing the systematic errors that the same person makes when matching a visual position with that index finger, should allow one to predict the systematic errors that the person will make when matching the visual position with the other index finger. If one cannot, we would have to conclude that the map-based and posture-based explanations are not enough. This would mean that the matching errors depend on what is compared with what, leading us to the transformation-based explanation. According to that explanation, it might even matter whether you move your left index finger to the position of the right one or vice versa. We will therefore use various combinations of tasks to compare matching errors within and across modalities.

In this study, we present two experiments. First, we designed an experiment in which the same visual targets were matched in different ways: with the left or right hand, each in two very different postures (above and below a surface). According to the map-based explanation, we would expect to see a consistent difference between the two arms irrespective of their posture. According to the posture-based explanation, we might expect similar (perhaps mirror-symmetric) visuo-haptic matching errors for both hands when the posture of both arms is similar, and different visuo-haptic matching errors for a single arm when its posture is varied. In the second experiment, we compared the matching errors across sets of tasks that ended in the same configuration (both in terms of visual location and of the arm and its posture), but that involved different actions to reach that configuration. For example, we compared bringing the index finger to a visual target dot with bringing a visual dot to the index finger.

## Experiment 1

In “Experiment 1” section, we explored the visuo-haptic matching errors for the right and the left hand when reaching for visual targets with different postures.

### Methods

#### Subjects

Ten self-reported right-handed subjects (6 female; 26–56 years of age) participated in the experiment, including two of the authors (MG and EB). Except for the authors, all subjects were naive about the purpose of the experiment. All subjects participated voluntarily, had (corrected-to-) normal vision, and gave their written informed consent. The experiment was part of an ongoing research program that has been approved by the ethics committee of the Faculty of Human Movement Sciences of Vrije Universiteit Amsterdam.

#### Stimulus and apparatus

**Fig. 1:**
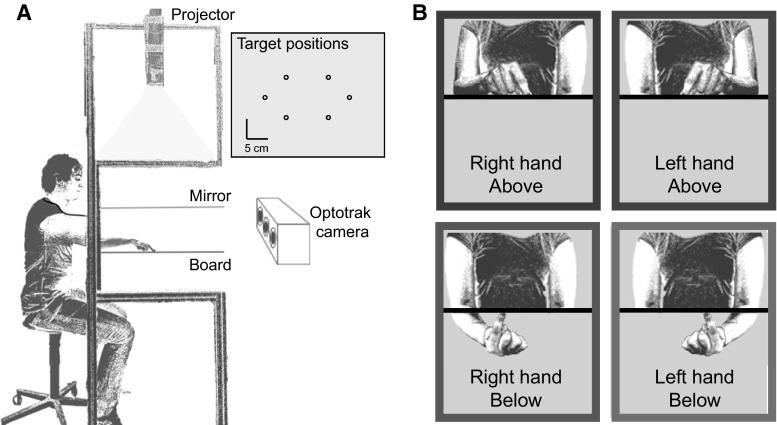
Setup. **a** The experimental setup. Visual targets were projected onto a surface above a mirror, so that subjects saw this surface’s reflection below the mirror (at the height of the board) but could not see their hand. The *inset* shows the six target positions as presented on the surface. **b** View of the four different matching tasks in “Experiment 1” section from the vantage point of the Optotrak; each hand (*right* and *left*) moved in two different postures (*above* and *below* the board) to the same visual targets

We used the same setup as we used previously to measure visuo-haptic matching errors (Kuling et al. 2013, 2015, 2016). In this setup, visual targets (15-mm-diameter dots) could be projected onto a horizontal screen above a (semi-silvered) mirror (Fig. 1a). We used six different target positions (as in Kuling et al. 2014, 2016). A thin board (5 mm) was placed at the same height as the apparent height of the projection plane as seen through the mirror, so the targets that were projected onto the screen appeared to lie on the thin board. During the experiment, the subject could not see his arms and hands because they were in the dark below the mirror. The area between the subject and the setup was covered with a dark cloth, so the subjects could not see their arms.

The position of the index finger was recorded with an Optotrak 3020 system (Northern Digital, Waterloo, Canada) at a sampling rate of 200 Hz. To do so, an infrared-emitting diode was attached to the nail of the index finger. Before each block of trials, we turned on the light below the semi-silvered mirror so that both the finger and target were visible and asked subjects to move to four dots in the four corners of the set up. This calibration allowed us to align the Optotrak coordinates of the index finger with the coordinates of the visual presentations.

Subjects moved their unseen index finger to visually displayed targets. This task was done in four different ways: all combinations of the right and left hand above and below the board. Four more ways of performing the task (involving passive matching) were presented in the same session, but not analyzed for this paper. An illustration of the tasks can be seen in Fig. 1b.

#### Procedure

Each matching task was presented as a separate block of 60 trials (ten for each of the six targets). The blocks were presented to the subjects in counterbalanced order. The order of the trials was semi-random: The six target positions were presented once, in a random order, before a next set of six targets was presented in a new random order. Since subjects moved from target to target, we ensured that the first target of a set was never identical to the last one of the previous set. Subjects did not receive any feedback during the experiment other than from their own proprioception. Previous studies have shown that there is little drift or accumulation of errors in this paradigm (Kuling et al. 2013, 2015). Van den Dobbelsteen et al. (2001) showed that reaching for visual targets is mainly guided by the intended end point (end point coding). Consequently, the end points in the present study should not depend much on the starting point (or direction of motion). Moreover, by averaging across various starting positions for the same target we would remove any residual biases related to the direction of movement on individual trials (although such influences would contribute to estimates of the trial-by-trial variability).

Subjects received verbal instructions about the task before the start of a block. They had to move the unseen index finger of the indicated hand (left or right) in the indicated posture (above or below the board) to the visually presented dot in one continuous movement. Then, the next dot appeared and the subject reached for it in the same way. This continued until the whole block was completed.

#### Analysis

The end of a movement was detected online and used to stop the trial and start the next one. We started looking for the end after the movement had reached a velocity of 50 cm/s. The end point was then defined as the position of the marker at the first frame after it had moved slower than a threshold of 3.5 cm/s for eight frames.

For each subject, block and trial (*i*) we calculated the matching error (ME), which is the vector between the target position in that trial (*T*_*i*_) and the end point of the finger movement toward this target (*X*_*i*_).

1 $$\overline{\text{ME}}_{i} = X_{i} - T_{i}$$

**Fig. 2:**
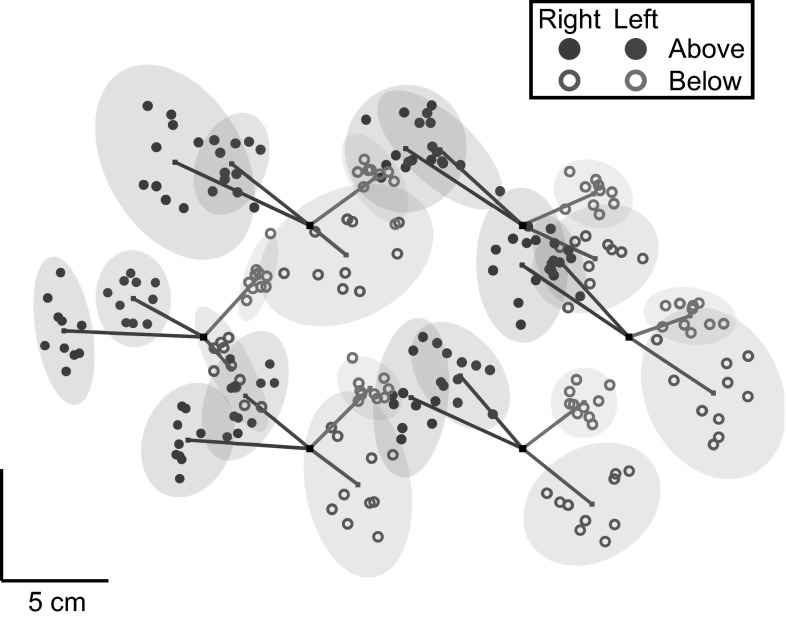
Results of one of the subjects in “Experiment 1” section. The *dots* show the end points for each trial. The *lines* show the mean matching errors, and the *ellipses* are 95% confidence *ellipses* for the distributions for the six targets and four matching tasks. This subject shows substantial matching errors that differ clearly between the four matching tasks. The average matching error for this subject is 4.3 cm (range 2.0–7.0 cm); the average area of the confidence *ellipses* is 18 cm^2^ (range 4.2–47.4 cm^2^)

Furthermore, the 95% confidence ellipses of the distribution of end points (this is the smallest possible ellipse that would be expected to contain 95% of the end points of such movements assuming that the measured points are normally distributed) were calculated for each subject, block and target; we refer to this measure as matching variability. The matching errors and matching variability of an example subject are shown in Fig. 2. The magnitude (Euclidian norm) of the matching errors and the area of the confidence ellipse were compared between hands, postures and target positions with 2 × 2 × 6 repeated measures ANOVAs. Greenhouse–Geisser corrections were used when sphericity was violated.

As in Kuling et al. (2016), a consistency value (CONS) was used to determine whether the matching errors were consistent across blocks. The consistency value incorporates both the magnitude and the direction of the matching errors by dividing the length of the difference vector between two matching errors by the sum of the lengths of the two matching errors. A value of 0 would indicate identical matching errors; a value of 1 would indicate matching errors in opposite directions. To turn this value into an intuitive indicator of consistency, whereby a larger value corresponds with more consistent errors, we subtract it from 1. It is defined as

2 $${\text{CONS}}\left( {\overline{\text{ME}}_{1} ,\overline{\text{ME}}_{2} } \right) = 1 - \frac{{\left| { \overline{\text{ME}}_{1} - \overline{\text{ME}}_{2} } \right|}}{{\left| {\overline{\text{ME}}_{1} } \right| + \left| {\overline{\text{ME}}_{2} } \right|}}$$

with ME_1_ and ME_2_ being vectors as defined in Eq. (1).

Equation (2) can be used to quantify the consistency between any two vectors. It can therefore provide a measure for the consistency between a single subject’s matching errors on two single movements to a single target, but it can also (after appropriate averaging of the ME’s) provide a measure for the consistency between the mean matching errors of two subjects for movements to that target. We determined the consistency for various comparisons.

The consistency value was calculated separately for the matching errors of each subject *s* $\left( {{\text{CONS}}_{\text{individual}}^{s} } \right)$, based on the mean matching error for each target *t* in the two series of trials that are being compared:

3 $${\text{CONS}}_{\text{individual}}^{s} = \frac{1}{6}\sum\limits_{t = 1}^{6} {{\text{CONS}}\left( {\overline{\text{ME}}_{t,s}^{{{\text{series}}1}} ,\overline{\text{ME}}_{t,s}^{{{\text{series}}2}} } \right)}$$

This was done separately for four comparisons: the consistency between hands for each posture, and the consistency between postures for each hand.

To give more meaning to the consistency value, we compared it to an estimate of the minimum expected value (the consistency between each subject and all other subjects: ${\text{CONS}}_{\text{others}}^{s}$), which we defined as the consistency value when matching the mean errors for the same task performed by different subjects, averaged across all targets (Eq. 4).

4 $${\text{CONS}}_{\text{others}}^{s} = \frac{1}{54}\sum\limits_{t = 1}^{6} {\sum\limits_{k = 1, \,k \ne s}^{10} {{\text{CONS}}\left( {\overline{\text{ME}}_{t, k} ,\overline{\text{ME}}_{t, s} } \right)} }$$

The indices *k* and *s* represent the ten subjects, so the comparison is between the mean data of one subject for a specific target and the mean data of each of the other subjects for the same target. We also estimated the value that one would expect on the basis of the trial-by-trial variability if the systematic errors were actually the same in the two cases by calculating the mean of the consistency values for all possible comparisons between the single trials of a subject within a task for each target (${\text{CONS}}_{\text{var}}^{s}$). CONS_var_ is an estimate of the consistency value you can expect based on the trial-by-trial variability, i.e., the value that corresponds to a consistent matching error considering the variability across trials. It was averaged over targets (Eq. 5)

5 $${\text{CONS}}_{\text{var}}^{s} = \frac{1}{540}\sum\limits_{t = 1}^{6} {\sum\limits_{i = 1}^{10} {\sum\limits_{j = 1,\,j \ne i}^{10} {{\text{CONS}}\left( {\overline{\text{ME}}_{t,s}^{i} ,\overline{\text{ME}}_{t,s}^{j} } \right)} } }$$

The indices *i* and *j* refer to the ten individual trials, so this measure compares all possible pairs of trials for a specific target within a specific series of trials. To get a value of CONS_var_ for a comparison between series of trials, we first calculated the CONS_var_ for the series of trials that are in the comparison and then average across them.

Since we are interested in the consistency between the postures of the two hands and the two hands in both postures, we calculate CONS_individual_, CONS_others_ and CONS_var_ for these four combinations. For the comparisons across postures and hands, two 3 × 2 repeated measures ANOVAs (Consistency type × posture/hand) were used to determine how the three consistency measures related to each other (in particular how CONS_individual_ relates to CONS_others_ and CONS_var_) for the two postures and the two hands, respectively.

### Results

**Fig. 3:**
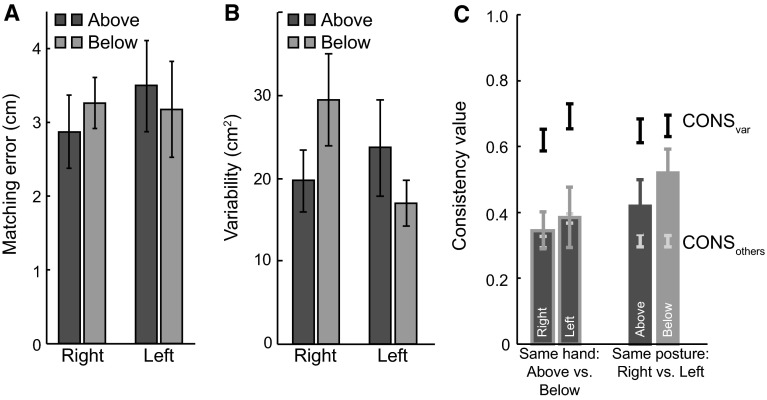
Results of “Experiment 1” section. **a** The mean matching error for each of the four matching tasks. **b** Mean matching variability for the four matching tasks. **c** The consistency of individual matching errors across postures and hands. The *bars* indicate CONS_individual_: On the *left*, the comparisons are shown for the same hand in different postures, and on the *right* for the same posture with different hands. In all panels, *error bars* show the SEM across subjects

The magnitudes of the matching errors ranged from 0.9 to 10.1 cm across subjects, target positions and tasks. The 2 × 2 × 6 repeated measures ANOVA on the magnitude of the matching errors showed no significant main effects of hand, posture or target position (all *p*’s > 0.25), and there were no significant interactions (all *p*’s > 0.11; Fig. 3a).

The 2 × 2 × 6 repeated measures ANOVA on the matching variability (range 7.0–72.1 cm^2^) showed no significant main effects (all, *p*’s > 0.41). The interactions were also not significant (all *p*’s > 0.18), except for a significant interaction between hand and posture (*F*_1.0, 9.0_ = 9.2, *p* = .014). The matching variability was larger for the right hand when it was below the board and for the left hand when it was above the board (Fig. 3b). This pattern is also visible in the example (Fig. 2): Blue open circles are more variable than blue filled circles, whereas for the green ones, the filled circles are more variable. We observed a similar trend (that is not significant) in the mean matching error: a larger systematic error for left above and right below (Fig. 3a).

The 3 × 2 repeated measures ANOVA on the consistency between moving above and below the board (left side of Fig. 3c) showed a main effect of Consistency type (*F*_1.2, 10.5_ = 24.2, *p* < .001) with no effect of hand or interaction (both *p*’s > .34). Post hoc pairwise *t* tests with Bonferroni corrections show that CONS_individual_ and CONS_others_ are significantly smaller than CONS_var_ (*p* = .005, and *p* < .001, respectively), whereas CONS_individual_ does not differ significantly from CONS_others_ (*p* = 1.0). Thus, there appears to be very little consistency between the matching errors when moving above and below the board.

The 3 × 2 repeated measures ANOVA on the consistency between the left and right hand (right side of Fig. 3c), showed a main effect of Consistency type (*F*_1.2, 11.0_ = 32.3, *p* < .001), with no effect of posture or interaction (both *p*’s > .22). Post hoc comparisons with Bonferroni corrections show that CONS_individual_ and CONS_others_ are significantly smaller than CONS_var_ (*p* = .005, and *p* < .001, respectively). The tendency for CONS_individual_ to be larger than CONS_others_ was not significant (*p* = .078). Thus, there also appears to be very little consistency in the matching errors between the hands.

In summary, the errors made in the two postures of the same hand are very different, even tending to be more different than the errors made by the different hands in the same posture. It is thus clear that we cannot speak of a single map of haptic locations of each hand.

**Fig. 4:**
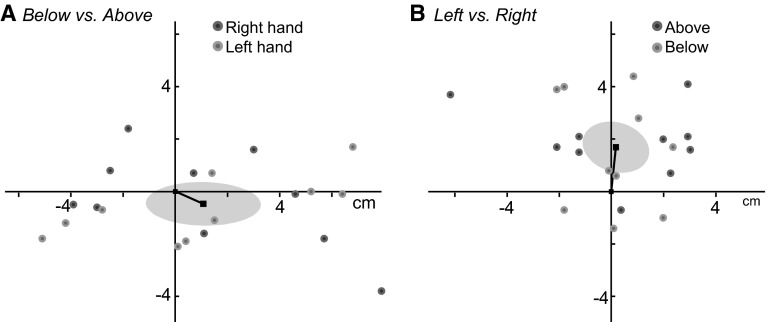
Differences between mean end points of the hand in the different matching tasks (in cm). *Each dot* represents the average difference between two tasks for a single subject. The *ellipses* show the 95% confidence interval of the mean of all data points. **a** The matching errors for reaching *below* the board compared to reaching *above* the board. **b** The matching errors for reaching with the left hand compared to reaching with the right hand. Subjects reached further away from the body when matching with their left hand compared to matching with their right hand

That the matches tended to be more consistent for the same posture made us wonder whether the lack of consistency could be explained by biomechanical factors. If the lack of consistency in matching errors were due to the biomechanics of the required movements, one would expect that the differences between the mean end points for the two postures would be consistent in direction across subjects. To examine this, we plotted the individual errors in Fig. 4a. The differences are plotted relative to the reached position above the board. The square shows the mean position of the dots, which is the mean position of the finger below the board relative to the one above the board, and the ellipse shows the 95% confidence ellipse for this mean. Since the origin is located within the ellipse, the systematic differences in matching error between reaching from above and below the board are idiosyncratic rather than systematic across subjects. The mean differences between the end points for the two hands for each subject and posture are plotted in Fig. 4b. A systematic shift of the reached positions can be seen: The origin (position of the right hand) is not located within the ellipse (95% confidence interval of the mean relative position of the left hand). This shows that on average subjects moved to a position further from their body when moving their left index finger than when moving their right index finger (to the same visual targets).

### Discussion

The goal of the first experiment was to determine whether visuo-haptic matching errors could be explained by a map-based approach or whether the posture had to be considered. The results show very little consistency between the matching errors in different postures, even less than the consistency when matching in the same posture with different hands (Fig. 3c). The consistency values for both types of comparisons were low compared to the consistency values estimated on the basis of the variability between trials (CONS_var_) and much lower than we found for repeating the same task in our previous study (Kuling et al. 2016).

The low consistency values for comparing postures mean that posture influences matching, which means that there cannot be a single haptic map for each hand. Therefore, we can reject map-based explanations of the matching errors. This result is consistent with the results of Rossetti et al. (1994), who found systematic changes in the matching error between the two hands when the posture of one of them was changed. The results are also in line with the results of our previous study (Kuling et al. 2014), in which we compared matching errors between pointing with the index finger and with the handle of a device that was held in a power grip. In that study, we found that the precision and the accuracy of the two pointing tasks were similar, but the matching errors were not consistent.

From the current results, we cannot fully reject the option of a posture-based explanation. The consistency between the errors for the two hands in the same posture tends to be larger than between subjects, supporting such an explanation, but the finding that this consistency is much smaller than CONS_var_ questions this posture-based explanation. Our second experiment compares the posture-based explanation with the transformation-based explanation.

## Experiment 2

To examine whether transformation-based explanations are required to account for systematic matching errors, we designed tasks that are based on the same set of matching configurations (both in terms of visual locations and of the arm and its posture), but that would be likely to involve different sensory transformations. According to any model that assumes that matching errors are due to sensory biases [e.g., the component transformation model in Simani et al. (2007)], the way the configuration is reached should not matter. We reasoned that the information that is used to perform different tasks that end in the same configuration might be coded in different reference frames. We therefore compared complementary tasks that involve different actions to reach the same configuration. For example, we compared bringing the right index finger to a visual target dot (whereby the end point is coded in a visual reference frame, that is probably transformed into an egocentric position for guiding the hand) with bringing a visual dot toward the right index finger (whereby the end point is coded in the right arm’s reference frame and is probably transformed into a visual reference frame for guiding the dot’s movements).

Besides comparing reversed matches, we also compared combinations of matching errors that involved the same (or at least very similar) movements. In such cases, even if the systematic errors depend on the movements that are made, it should be possible to combine the matching errors of two tasks (e.g., moving the left and right index fingers to a visible dot) to predict the errors that will be found in a third task in which the common sensory information (in this example the visible dot) is eliminated (for instance by moving the left and right index fingers simultaneously to the same position). Therefore, we added haptic–haptic matching tasks to the visuo-haptic matching tasks that were used in “Experiment 1” section. For these tasks, there is no need to transform information from a visual to a haptic coordinate frame or vice versa. None of the explanations predict inconsistent errors in the latter tasks.

### Methods

#### Setup and procedure

**Fig. 5:**
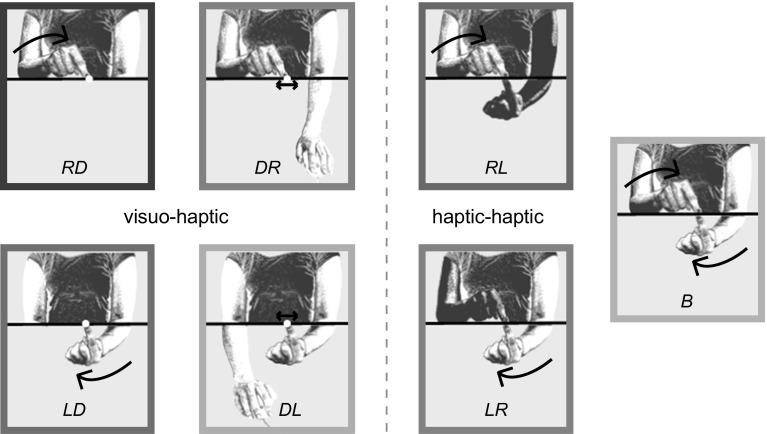
Seven different matching tasks in “Experiment 2” section. Four tasks were visuo-haptic: moving the unseen index finger to a visual target (*leftmost column*) or moving a visual target to the unseen index finger (*second column*). The other three tasks were haptic–haptic: matching the positions of both index fingers in different ways (*third and fourth column*). The *pink curved arrows* indicate the active matching arms; the *pink bi-directional arrows* indicate cursor-movement to match the static position of the hand (color figure online)

We used the same setup as in “Experiment 1” section. The same subjects participated. Seven tasks were presented to the subjects in separate blocks (Fig. 5). Two tasks were the same as two of the tasks in “Experiment 1” section: Subjects had to move to a visual target either with the index finger of their right hand above the board, or with the index finger of their left hand below the board. We will refer to these as RD (right to dot) and LD (left to dot). Two other tasks were designed to have the same set of matching configurations as the previous two, but for it to be reached in a different way: Subjects had to bring a visual dot either to the position of their right index finger that was above the board (DR, dot to right) or to the position of their left index finger that was below the board (DL, dot to left). Before the subjects moved the dot, we first guided the index finger in question to the target position. Twenty visual arrows were presented on the screen (four rows of five arrows, 1 cm apart, 20 cm further away from the center of the body than the center of the workspace), representing the vector from the index finger to the target. These arrows guided the subject’s unseen index finger toward the target location for that trial. Each of the visual arrows was ten times smaller than the distance between the index finger and the target. Once the index finger was within 2 mm of the center of the target, the arrows disappeared. This method is similar to the arrow method used by Sober and Sabes (2005), Cheng and Sabes (2007) and was also used in our previous study (Kuling et al. 2016). When the arrows disappeared, a visual dot appeared at the center of the grid of arrows. Subjects moved the dot with the other hand using a computer mouse and clicked the mouse-button once they thought the dot was at the same position as the index finger in question. The motion of the cursor dot was scaled by a factor 10 with respect to that of the mouse, to make sure that the distance that the mouse moved did not provide any useful information.

In addition to these four visuo-haptic matching tasks, we performed three haptic–haptic matching tasks. The same guidance of the target finger was used for two of these tasks as for the DR and DL tasks described above. Subjects then moved the index finger of their right hand above the board to match the position of the index finger of their left hand below the board (RL), or moved their left index finger to match the position of the right index finger (LR). The third haptic–haptic matching task was matching both index fingers at the same time (B). In this task, a large target area (diameter of 15 cm) was flashed (centered on one of the six target positions), and participants moved their two index fingers simultaneously to some position within that target area, with the fingers aligned on opposite sides of the board.

The seven tasks were performed in a single session. Their order was systematically varied across participants. Each task took approximately 5–7 min. After each task, there was a short break. Within each task, there were ten movements to each of the six targets, as in “Experiment 1” section.

#### Analysis

The end points of finger movements (in the RD, LD, LR, RL and B tasks) were determined in the same way as in “Experiment 1” section. The dot’s location (in the DR and DL tasks) was defined as the position of the cursor dot at the time of the mouse click; the measured position of the index finger at that moment was used as the target position.

For the four visuo-haptic matching tasks, the matching errors were calculated by comparing the position of the index finger with the position of the dot. For the haptic tasks, the matching errors were the differences between the positions of the active index finger with respect to the passive one. In the B task, in which both hands moved, we report the matching error as if the right index finger was the target position.

For the visuo-haptic matching tasks, two 2 × 2 × 6 repeated measures ANOVAs (finger × action × position) were used to determine whether the magnitudes of the matching errors and matching variability depended on the task (moving the index finger to the target dot or moving the cursor dot to the index finger) and on which index finger was used (left or right). For the haptic tasks, two 3 × 6 repeated measures ANOVAs (task × position) were used to determine whether the task (LR, RL or B) influenced the magnitude of the matching errors or their matching variability. If sphericity was violated, Greenhouse–Geisser corrections were used.

**Fig. 6:**
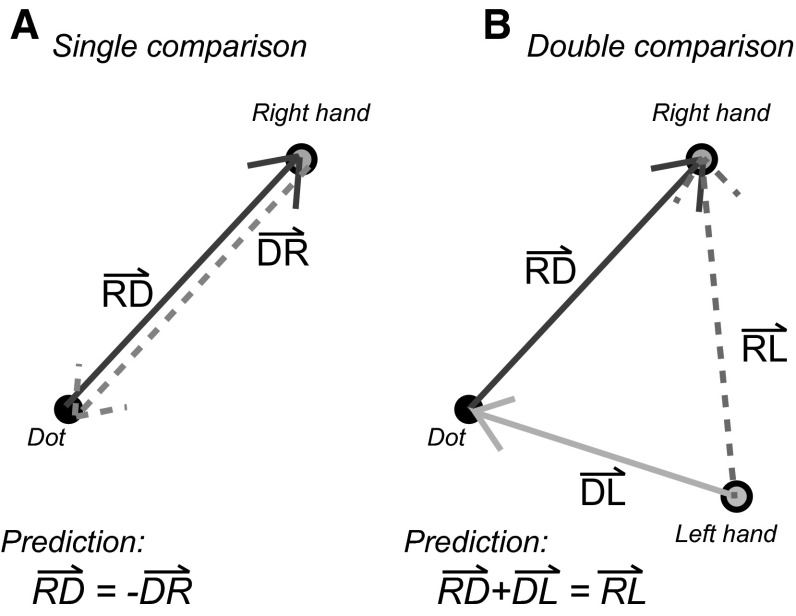
Examples of the expected outcome in the **a** single comparison predictions and **b** double comparison predictions according to the posture-based explanation. *Each arrow* represents the systematic error for one matching condition

As in “Experiment 1” section, we calculated consistency values between tasks and compared them to a minimum expected value (the consistency between subjects: CONS_others_) and an expected value based on the subjects’ trial-by-trial variability for each target within a task (CONS_var_). We calculated the consistency values between the tasks with the same matching configuration to test for the reversibility of sensory matching errors both within and between modalities; single comparisons (see Fig. 6a for an example). The single comparisons were made for: RD compared with −DR, LD compared with −DL, and all three combinations of −RL, LR and B. For the visuo-haptic comparisons, a 3 × 2 repeated measures ANOVA (Consistency type × hand) was used to determine how the individual consistency values (between moving the hand to the dot or the dot to the hand) related to CONS_others_ and CONS_var_ for the two hands. For the haptic–haptic comparisons, a 3 × 3 repeated measures ANOVA (Consistency type × hand order) was used to evaluate the same for all three possible pairs of matches.

To test whether sensory matching errors of two tasks can be combined to predict the outcome of a third task, we made several double comparisons (see Fig. 6b for an example). We compared matches with and without an intermediate value: RD + DL with RL, LD + DR with LR and RD + LD with −B. We also compared RD + LD with RL and with -LR and DR + DL with −B. If matching errors are based on sensory biases, one would expect a high consistency (close to CONS_var_) for all comparisons. A 3 × 6 repeated measures ANOVA (Consistency type × combination) was used to determine how the individual consistency values related to CONS_others_ and CONS_var_.

### Results

**Fig. 7:**
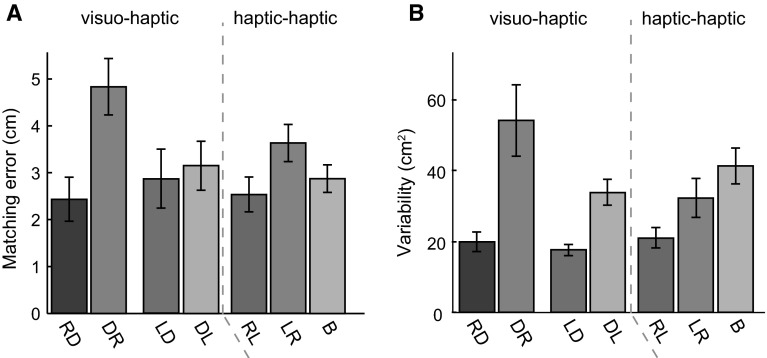
Results of “Experiment 2” section. **a** The mean matching error for the seven matching tasks. **b** Mean matching variability for the seven matching tasks. *Error bars* show the SEM across subjects

The magnitudes of the matching errors ranged from 0.1 to 10.1 cm across subjects, target positions and tasks. The mean errors for all tasks are shown in Fig. 7a. The RM ANOVA on the magnitudes of the matching errors in the four visuo-haptic matching tasks revealed a significant main effect of action (*F*_1.0, 9.0_ = 5.9, *p* = .039): The matching errors were larger when moving the dot to the finger than when moving the finger to the dot. There was no significant effect of hand used (*F*_1.0, 9.0_ = 1.4, *p* = .266). There was a significant effect of position (*F*_1.5, 13.9_ = 7.5, *p* = .009): As reported in the literature, the matching errors for positions closer to the body were smaller than those for positions further away (Van Beers et al. 1998; Kuling et al. 2014). There were also significant interactions between hand used and action (*F*_1.0, 9.0_ = 5.8, *p* = .040), and between hand used and target position (*F*_2.7, 24.1_ = 3.5, *p* = .036). The RM ANOVA on the magnitudes of the matching errors in the three haptic tasks showed a significant effect of task (*F*_2, 18_ = 7.4, *p* = .005): The errors were larger for LR compared to RL and B. There was also a significant effect of target position (*F*_5, 45_ = 4.3, *p* = .003).

The matching variability (surface areas of the 95% confidence ellipses, Fig. 7b) of the matched positions varied across subjects, target positions and tasks (range 4.4–210.0 cm^2^). The RM ANOVA on the matching variability for the four visuo-haptic matching tasks showed a significant main effect of task (*F*_1, 9_ = 31.4, *p* < .001): Moving the finger to a dot was more precise than moving the dot to the finger. There was also a main effect of finger used (*F*_1.9_ = 5.5, *p* = .044): The matching variability for the right index finger was slightly larger than that for the left index finger. Interaction effects of task and finger used (*F*_1, 9_ = 5.2, *p* = .049) and finger used and target position (*F*_5, 45_ = 2.9, *p* = .025) were also found, which could be explained by there being exceptionally large matching variability in DR for some of the target positions. The RM ANOVA on the matching variability in the haptic tasks showed a significant effect of task (*F*_1.2, 11.2_ = 5.9, *p* = .028). Post hoc comparisons with Bonferroni corrections show that B was more variable than RL (*p* = .001).

**Fig. 8:**
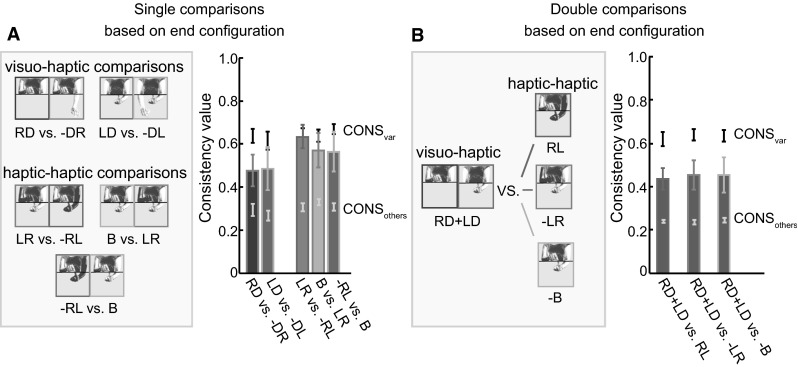
Consistency comparisons of “Experiment 2” section. **a** Consistency values for the single comparisons with the same matching configurations. All consistency values are higher than the values for random pairings across subjects (CONS_others_). The visuo-haptic comparisons are lower than the consistency estimated from the trial-by-trial variability (CONS_var_), while the haptic–haptic comparisons do not differ significantly from these estimates. **b** Consistency values for the double comparisons. All consistency values are higher than the values for random pairings across subjects (CONS_others_), but lower than the consistency estimated from the trial-by-trial variability (CONS_var_).

The aim of our analysis was to test whether we can explain the matching errors in terms of sensory biases. If so, we would expect that the errors in the two tasks that involved the same matching configuration would be consistent with each other. The consistency is expected to be about CONS_var_, much higher than CONS_others_. For the single comparisons in the visuo-haptic tasks (left two bars in Fig. 8a), the 3 × 2 RM ANOVA (Consistency type × hand) showed a main effect of Consistency type (*F*_1.2, 11.0_ = 30.2, *p* < .001), and no significant effect of hand or interaction effect (both *p*’s > .82). Post hoc comparisons with Bonferroni correction show that CONS_individual_ and CONS_others_ are significantly smaller than CONS_var_ (*p* = .024, and *p* < .001, respectively), and CONS_individual_ was significantly larger than CONS_others_ (*p* = .023).

For the single comparisons of the haptic–haptic tasks (right three bars in Fig. 8a), the 3 × 3 RM ANOVA (Consistency type × task) showed a main effect of Consistency type (*F*_1.2, 10.4_ = 23.6, *p* < .001), and no significant effect of task or interaction effect (both *p*’s > .34). Post hoc comparisons with Bonferroni correction show that CONS_others_ was significant smaller than CONS_var_ (*p* < .001) and CONS_individual_ was significantly larger than CONS_others_ (*p* = .007), but there was no significant difference between CONS_individual_ and CONS_var_ (*p* = .956).

For the double comparisons (Fig. 8b), the 3 × 6 RM ANOVA (Consistency type × task) showed a main effect of Consistency type (*F*_2, 18_ = 83.3, *p* < .001), and no significant effect of task or interaction effect (both *p*’s > .17). Post hoc comparisons with Bonferroni correction show that CONS_individual_ and CONS_others_ are significant smaller than CONS_var_ (*p* < .001, and *p* < .001, respectively), and CONS_individual_ was significantly larger than CONS_others_ (*p* = .006).

What is the cause of the lack of consistency that we find? Can it be explained by asymmetries between the tasks? It might for instance be that subjects prefer to minimize muscle activation, and therefore position an active finger closer to the body. To test whether the lack of consistency is due to such systematic differences between tasks, we compared the mean end points in similar tasks that differed in whether it was a certain index finger that was moving to the target during the matching process (we refer to such an index finger as the active finger). We compared the average bias for the active finger tasks (RD and LD) with that in the corresponding passive finger tasks (−DR and −DL). We did not find any systematic differences between the matching errors across subjects in any of the comparisons (not shown). This suggests that the difference between the errors that are made when moving the finger to the dot and moving the dot to the finger is idiosyncratic rather than being consistent across subjects. We also compared the average −RL, LR and B biases, because in LR the right hand could rest a bit on the table, while in the other tasks this hand was actively moving. Again, we did not find a systematic difference in matching errors across subjects, suggesting that the lack of consistency between moving the right to the left index finger and moving the left to the right finger is also due to idiosyncratic errors rather than ones that were consistent across subjects.

### Discussion

There is clearly some consistency between the errors that individual subjects made in the complementary tasks (Fig. 7a: All consistency values are larger than CONS_others_), but for all comparisons that involved matching haptics and vision, the consistency was less than we estimated on the basis of the trial-by-trial variability. If the biases had been completely consistent in the complementary tasks (i.e., what one would expect based on a posture-based explanation), we would have expected the consistency value to be higher than that of CONS_var_, because the consistency value is based on the mean error, while CONS_var_ is based on the error on individual trials, so the former is less susceptible to random variability across trials. Based on the single comparisons of visuo-haptic matching tasks, we can conclude that moving the index finger to a dot does not give rise to the same mismatch as moving the dot toward the index finger. Based on the double comparisons (Fig. 7b), we conclude that moving the right index finger to a visual dot and moving the left index finger to the same visual dot do not lead to the same mismatch as directly matching the positions of both fingers. For the haptic–haptic matching tasks, the errors were similar for complementary tasks: The errors when matching both index fingers in the same configuration do not depend on the order of the movements (right first, left first, both at the same time). This suggests that systematic visuo-haptic matching errors must have both posture-based and transformation-based components, while the systematic matching errors in the haptic–haptic task are mainly posture-based.

The matching variability of the dot to right (DR) task is higher than that in the other visuo-haptic matching tasks (Fig. 6). This might be related to the findings that the control of the dominant and non-dominant hand differ in many respects, and the suggestion that the position control might be more accurate for the non-dominant hand (Sainburg and Kalakanis 2000; Sainburg and Schaefer 2004). As all subjects were right-handed this would imply more matching variability when the right hand was the passive target. Alternatively, one might argue that subjects were not used to using the computer mouse with their left hand. However, considering that CONS_var_ is based on the trial-by-trial variability, and the consistency for the RD versus −DR comparison was significantly lower than this value, we do not think that the variability in using the computer mouse influenced our interpretation of the consistency values. Taken together, the results suggest that transformations between the modalities lead to errors that are not invertible. Perhaps errors are introduced when transforming visual information to haptic information, which are not the same as for the reversed transformation.

## General discussion

In this paper, we described two experiments that examined errors in aligning the positions of the hands to each other and to visually perceived positions. In the first experiment, we showed that the posture of the arm influences the matched position. The matching errors were also different for the two hands. These results are not in line with a map-based explanation of the matching errors. In the second experiment, we showed that even matching tasks that involve the same configurations of the arm and the visual target do not always lead to comparable errors when performed differently. Moving the index finger to a dot does not lead to the same mismatch as moving the dot toward the index finger. Neither does moving the right index finger to a visual dot and the left index finger to the same visual dot lead to the same mismatch as directly matching the positions of the two fingers. This means that the direction of the transformation of the information between modalities influences the errors that are made, presumably because the different transformations are conducted differently. Only moving the two index fingers to each other with different orders of the movements gives rise to similar mismatches.

Simani et al. (2007) tried to explain multisensory mismatches by summing the separate sensory and task-dependent components. They used a set of alignment tasks to study the aftereffects of visual shift adaptation (Van Beers et al. 1996, 2002). Their tasks were similar to our matching tasks (both visuo-haptic and haptic–haptic), but they introduced a visual shift and analyzed aftereffects of this manipulation, while we investigated the sensory matching errors themselves. They presented two models that might describe the underlying mechanisms of sensory coordination. In the component transformation model, a visually perceived position that is initially coded with respect to gaze is first transformed to a position with respect to the body and this body position is then transformed to a position with respect to the reaching arm. In the direct transformation model, the visual information is directly transformed to a position for the reaching arm. The observed aftereffects of adapting to a visual shift could best be explained by the component transformation model. Our data cannot distinguish between the two transformation models they compared. We found that the matching errors (and therefore the transformations) between the two hands are consistent (right three bars of Fig. 7a), which is in line with both models. On the other hand, we found that moving an unseen index finger to a visual dot does not lead to the same matching error as moving the visual dot the unseen index finger (Fig. 7b), which is in conflict with both models. The models should therefore be modified to include the direction of transformations, distinguishing between the eye-to-body and body-to-eye transformation in the component transformation model, and the direct eye-to-arm and arm-to-eye transformations in the direct transformation model.

Our results are thus not consistent with the hypothesis that the matching errors are simply combined sensory biases (irrespective of whether map-based or posture-based). This finding has important implications for our understanding of the principles by which the brain solves sensorimotor tasks. Apparently, the sensory errors are partly the result of the process of transforming information and sensory representation that depend on the planned action of getting to the target, instead of only being based on evaluating the desired configuration.

One could question whether the results are an artifact of a possible distinction between active and passive haptic position sense. It has been suggested that there is a drift in the perceived position of the hand when holding it at the same physical position for a long period of time (Wann and Ibrahim 1992). However, these shifts are small compared to the overall errors that we found. As the proprioception of the arm can drift in the DR and DL tasks, but not in the RD and LD tasks, we cannot fully exclude a shift due to drift of the target in the RD versus −DR and LD versus −DL comparisons. However, we do not see systematic differences between the matching in −RL versus LR, where the target finger is also in place for some time before the other arm moves, and the haptic comparisons with B, a condition without a static hand. Although in these cases the delay between the target finger arriving and the other finger reaching the target may be shorter than when matching with the dot, because bringing the dot to the target takes longer than moving one’s finger to the target, in the double comparisons (specifically RD + LD vs. −B) none of the tasks involve waiting with a target finger in place, and we still see large differences in matching.

To conclude, we found that sensory matching errors are not simply based on summing sensory biases. Transformations between modalities seem to play an important role, and the transformation seems to depend on the movement that needs to be made.
